# Important factors for achieving survival of five years or more in non-small cell lung cancer patients with distant metastasis

**DOI:** 10.3892/ol.2014.2107

**Published:** 2014-04-30

**Authors:** TOMONORI HIRASHIMA, HIDEKAZU SUZUKI, NORIO OKAMOTO, NAOKO MORISHITA, TADAHIRO YAMADORI, MOTOHIRO TAMIYA, TAKAYUKI SHIROYAMA, KANAKO KURATA, ICHIRO KAWASE

**Affiliations:** Department of Thoracic Malignancy, Osaka Prefectural Medical Center for Respiratory and Allergic Diseases, Habikino-shi, Osaka 583-8588, Japan

**Keywords:** non-small-cell lung cancer, prognostic factors, ≥5 years survival, survival post-progression

## Abstract

In order to examine which factors were important for achieving a ≥5 year survival time in non-small cell lung cancer (NSCLC) patients with distant metastasis, 268 NSCLC patients who received first-line chemotherapy between January 2004 and December 2007 were retrospectively examined. The median survival time of the patients was 14 months, with 22 surviving for ≥5 years, 48 for ≥2 years, but <5 years, and 198 surviving <2 years. Multivariate analysis determined that never having smoked, a good performance status, relapse following thoracic surgery and intra-thoracic metastasis were significantly favorable prognostic factors, while abdominal metastasis was a significantly poor prognostic factor. The ≥5 years and ≥2–5 years groups had significantly more favorable prognostic factors than the <2 years group. The never-smoked status was a particularly important factor for ≥5 years of survival. The ≥5 years and ≥2–5 years groups achieved a significantly more favorable response to first-line chemotherapy, and a greater number of regimens, total months of epidermal growth factor receptor (EGFR)-tyrosine kinase inhibitor (TKI) treatment and cytotoxic agent treatment cycles compared with the <2 years group. In total, ~50% of the patients received palliative radiotherapy. In the ≥5 years group, patients with EGFR drug-sensitive mutations achieved ≥5 years of survival mainly by EGFR-TKI therapy, while those without EGFR mutations achieved ≥5 years of survival by continuing effective cytotoxic agents. Achievement of >5 years of survival was found to correlate with the presence of favorable prognostic factors, response to first-line chemotherapy, provision of appropriate EGFR-TKI therapy according to genetic testing results, continuing effective cytotoxic regimens and the use of radiotherapy as local therapy.

## Introduction

The prognosis of patients with advanced non-small cell lung cancer (NSCLC), particularly the non-squamous subtype, could be improved. Studies by Mitsudomi *et al* (1) and Maemond *et al* (2) found that patients with lung cancer who were selected by epidermal growth factor receptor (EGFR) mutations have a significantly longer progression-free survival rate if they are treated with gefitinib compared with treatment with cisplatin-docetaxel or carboplatin-paclitaxel. Recent studies (3,4) have shown that the overall survival (OS) time in two former Japanese studies (1,2) was ≥2 years in the gefitinib and platinum doublet groups.

While several studies (5–9) retrospectively defined a ≥2-year survival time in patients with advanced NSCLC as long-term survival, however, recently, other patients with advanced NSCLC have been encountered who have survived ≥5 years (10). Kaira *et al* (11) also reported the survival of patients with advanced NSCLC for ≥5 years.

In the present study, the factors associated with extended survival time were examined along with the clinical features, types of treatments administered and EGFR mutations in patients with metastatic NSCLC who had survived for ≥5 years.

## Patients and methods

### Study approval

The present retrospective study was approved by the Institutional Review Board of the Osaka Prefectural Medical Center for Respiratory and Allergic Diseases (Osaka, Japan) on June 19, 2012 (approval no. 596).

### Criteria

Of the 360 patients with NSCLC in our previous study (10), those patients with stage IIIA cancer (n=27), relapse following chemoradiation therapy (n=22) or thoracic radiation therapy (n=2), local recurrence following thoracic surgery (n=5) and stage IIIB cancer without pleural disseminations or malignant pleural effusion (n=36) were excluded from the current study. Patients with pleural dissemination or malignant pleural effusion were considered as stage IV according the seventh edition of the tumor-node-metastasis (TNM) classification of the International Association for the Study of Lung Cancer (12). Overall, the clinical records of 268 NSCLC patients with distant metastasis who underwent first-line chemotherapy between January 1, 2004, and December 31, 2007, and had been observed for ≥5 years as of December 31, 2012, were retrospectively examined. For inclusion in the current study, all patients met the following criteria: A diagnosis of histopathologically-confirmed primary NSCLC (adenocarcinoma, squamous cell carcinoma, large cell carcinoma or unclassified NSCLC), the presence of distant metastasis at the time of diagnosis or recurrence following thoracic surgery according to the seventh edition of the TNM Classification of the International Association for the Study of Lung Cancer (12), treatment with systemic cytotoxic agents, either cytotoxic chemotherapy or EGFR-tyrosine kinase inhibitor (TKI) therapy, and perioperative chemotherapy that was not regarded as first-line chemotherapy. In the present study, unilateral multiple lung metastases were defined as distant metastasis.

### Baseline demographic information

Baseline demographic data, including gender, age, Eastern Cooperative Oncology Group performance status (PS), histology, stage and smoking status at the initiation of first-line chemotherapy, were obtained for each patient. At the time of analysis, the following data were available for all patients: A complete patient history with results from a physical examination, surgical and pathological reports, the results of mediastinoscopy, fiberoptic bronchoscopy, thoracoscopy and imaging (chest radiography and computed tomography or magnetic resonance imaging, and bone scintigraphy or positron emission tomography) and the number of metastatic organs and metastatic sites.

### Best response to first-line chemotherapy

The best response to first-line chemotherapy was determined by a review of the patient records compiled by physicians during weekly cancer conferences held at the Osaka Prefectural Medical Center for Respiratory and Allergic Diseases and based upon the Response Evaluation Criteria in Solid Tumors (RECIST) (13). Based on RECIST, the tumor response to cytotoxic agents was categorized as a partial response (PR), stable disease (SD) and progressive disease (PD). A tumor response that could not be evaluated was described as not evaluable (NE).

### Treatment

In addition to the data from our previous study (10), the data regarding the subsequent treatment of each patient were added to the database, including those regarding regimen, number of cycles, dosage and date of regimen initiation and termination.

### Palliative radiotherapy (RT)

While the palliative RT administered at the Osaka Prefectural Medical Center for Respiratory and Allergic Diseases had consisted of RT to the bone, whole brain and other sites, palliative RT at other institutions had consisted of stereotactic radiosurgery (SRS) to the brain metastases. All relevant data were collected from the patient clinical records.

### Survival

Survival time was defined as the period from the date of initiation of first-line chemotherapy to the date of mortality or last follow-up, and survival data were last updated on December 31, 2012. Subsequent to the estimation of the survival rates using the Kaplan-Meier method, the patients were classified into three groups for comparison of their demographic and clinical characteristics as follows: Those who had survived ≥5 years (≥5 years group), those who had survived ≥2 years, but <5 years (≥2–5 years group) and those who had survived <2 years (<2 years group).

### Genetic testing for EGFR and other NSCLC-driving mutations

Tissue samples from surviving patients with NSCLC in the present study were sent to Mitsubishi Chemical Medience Corporation (Tokyo, Japan) for peptide nucleic acid-locked nucleic acid polymerase chain reaction clamp-based testing between June 1, 2007, and December 31, 2012. Per the protocol of the previous WJTOG 3405 (1) and WJTOG 0403 (14) clinical trials at the Osaka Prefectural Medical Center for Respiratory and Allergic Diseases, screening for drug-sensitive EGFR mutations, including a deletion in exon 19 or an L858R substitution in exon 21, was conducted. Additionally, the anaplastic lymphoma kinase (ALK) translocations were determined via fluorescence *in situ* hybridization and K-ras mutations as driver mutations in patients who had survived ≥5 years.

### Statistical analysis

Statistical analyses were performed using software package R (15). Patient background data were compared using the χ^2^ test and Fisher’s exact test for categorical factors. Patient treatment data were compared using the Mann-Whitney U test. P<0.05 was considered to indicate a statistically significant difference. Prognostic factors were analyzed by univariate and multivariate analyses using the Cox proportional hazards model. Differences among survival curves were assessed using the log-rank test. Multivariate analysis was performed among selected prognostic factors when P<0.005 in univariate analysis. A small number of significant prognostic factors in univariate analysis were excluded from multivariate analysis.

## Results

### Overall survival

**Figure 1:**
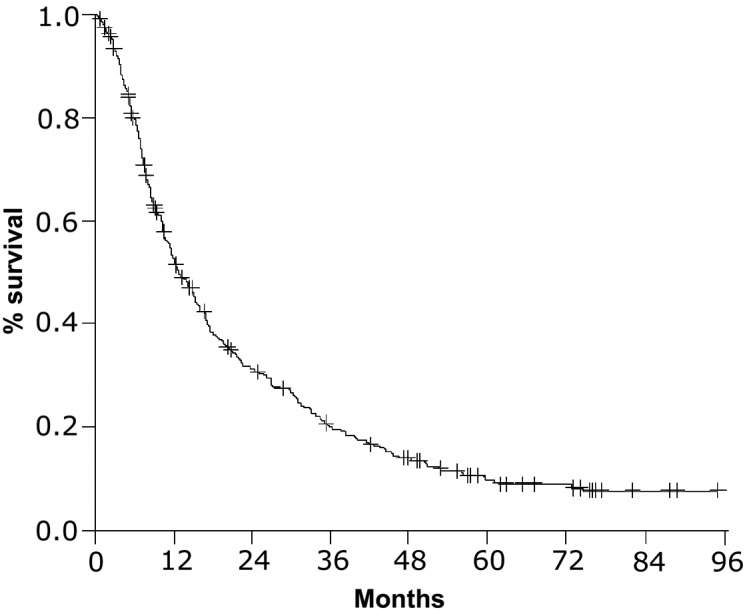
Survival curve of the 268 NSCLC patients with distant metastasis who had undergone first-line chemotherapy between January 1, 2004, and December 31, 2007. The median survival time (MST) was 14 months (95% confidence interval, 11.4–15.3 months). NSCLC, non-small cell lung cancer.

As shown in Fig. 1, the median survival time (MST) of the 268 NSCLC patients with distant metastasis examined in the present study was 14 months (95% confidence interval, 11.4–15.3). Based on a review of the survival data, 22 patients (8.2%) were placed in the ≥5 years group, 48 patients (17.9%) were placed in the ≥2–5 years group and 198 patients (73.9%) were placed in the <2 years group.

### Prognostic factors

As shown in Table I, by univariate analysis, gender, smoking status, PS, histology, relapse following thoracic surgery and number of metastatic organs were identified as significant prognostic factors (P=0.0003, P<0.0001, P<0.0001, P=0.0091, P=0.0017 and P=0.0460, respectively). The patients with metastasis to the intra-thoracic organs, including lungs, pleura and pericardium, had a significantly improved prognosis compared with those with other types of metastasis (P=0.0007). By contrast, the patients with metastasis to the abdominal organs, including adrenal glands, liver, kidney and other sites, had a significantly worse prognosis than those with other types of metastasis (P<0.0001). Multivariate analysis was performed among selected prognostic factors, including gender, smoking status, PS, relapse following thoracic surgery, metastasis to intra-thoracic organs and metastasis to abdominal organs in univariate analysis, which were confirmed as significant prognostic factors (P=0.0009, P<0.0001, P<0.0001, P=0.0009 and P=0.0006, respectively).

### Demographic characteristics according to survival duration

Table II summarizes the patient demographic characteristics of the three survival groups according to gender, histology and the significant prognostic factors determined by multivariate analysis. All 22 patients in the ≥5 years group, 41 out of 48 patients in the ≥2–5 years group and 157 out of 198 patients in the <2 years group had histologically-confirmed adenocarcinoma. Although the demographical data of the patients in the ≥5 and ≥2–5 years groups were extremely similar, analysis revealed a significantly greater number of patients that had never smoked in the ≥5 years group when compared with the ≥2–5 years group (P=0.0020). By contrast, the variables of female gender, never smoking, PS, relapse following thoracic surgery and metastasis to intra-thoracic organs were significant in the ≥5 years group when compared with the <2 years group (P=0.0017, P<0.0001, P=0.0158, P=0.0200 and P=0.0220, respectively). A significantly greater number of patients with a PS of 0 or 1 who had relapsed following thoracic surgery experienced metastasis to the intra-thoracic organs, and a significantly smaller number of patients who experienced abdominal metastasis were found in the ≥2–5 years group compared with the <2 years group (P=0.0269, P=0.0008, P=0.0469 and P=0.0133, respectively).

A total of 55 (20.5%) patients underwent EGFR mutation testing. Of these patients, 30 had wild-type EGFR, 21 had drug-sensitive mutations (18 with a deletion in exon 19 and three with the L858R mutation), and four had other types of mutations, consisting of one with a deletion in exon 19 plus a T790M mutation, one with a L861Q mutation in exon 21 and two with a point mutation in exon 18. The remaining patients were unable to undergo testing due to the inability to obtain either insurance approval or an adequate tissue sample.

### Treatment efficacy, EGFR-TKI therapy duration and number of cycles of cytotoxic agent therapy according to survival duration

Of the 268 patients, 165 (61.6%) underwent at least second-line chemotherapy, 96 (35.8%) at least third-line chemotherapy and 54 (20.1%) underwent at least fourth-line chemotherapy. As shown in Table III, compared with the patients in the <2 years group, the patients in the ≥5 years and ≥2–5 years groups experienced a PR or SD significantly more frequently (P=0.0011 and P=0.0002, respectively), underwent a significantly greater number of regimens (P<0.0001 and P<0.0001, respectively) and underwent EGFR-TKI therapy (63.6% of the ≥5 years group and 56.2% of the ≥2–5 years group vs. 29.8% of the <2 years group) significantly more frequently (P=0.0014 and P=0.0006, respectively). Patients in the ≥5 years group underwent significantly more total months of EGFR-TKIs treatment in the ≥2–5 years and <2 years groups (P=0.0068 and P<0.0001, respectively) and significantly more cycles of cytotoxic agent therapy (P=0.0045, and P<0.0001, respectively). By contrast, compared with the patients in the <2 years group, those in the ≥2–5 years group underwent significantly more total months of EGFR-TKIs treatment and cycles of cytotoxic agent therapy (P=0.0003 and P<0.0001, respectively). As shown in Table III, 142 (53.0%) of the 268 patients underwent palliative RT, including SRS to brain metastases, whole brain RT, RT to bone metastasis and RT to other metastatic sites. No significant differences were found among the three survival groups regarding frequency of RT to each site.

### Driver mutations in and treatment administered to the ≥5 years group

As summarized in Table IV, the 22 patients in the ≥5 years group were classified into four groups (A-D) according to their response to EGFR-TKIs and cytotoxic agent therapy. Five patients (group A) responded mainly to EGFR-TKI therapy, five patients (group B) responded to EGFR-TKI and cytotoxic agent therapy and eight patients (group C) responded mainly to cytotoxic agent therapy. Four patients (group D) exhibited indolent tumors and did not undergo cytotoxic agent therapy in the long term. Of the 17 patients in the ≥5 years group who had undergone driver mutation testing, one tested positive for ALK translocation. Seven patients tested negative for EGFR mutations, ALK translocation or K-ras mutations.

## Discussion

In a study by Van Damme *et al* (9), long-term survivors were defined as patients who survived >2 years, and it was concluded that there are extremely few clinical factors at the time of diagnosis that can distinguish survivors of >2 years from survivors of ≤2 years. Compared with these results, the present study showed PS, relapse following thoracic surgery and metastatic sites as significant prognostic factors, which distinguished the ≥2–5 years group from the <2 years group. For achieving ≥5 years of survival, the never-smoked status characteristic was the most significant prognostic factor in the present study. Furthermore, in a study of 112 patients who had experienced recurrence following surgery, Yamazaki *et al* (16) reported a good prognosis (MST, 25.6 months) and identified the significant prognostic factors to be histology, age, PS and abdominal metastasis. In this study and the present study, patients with abdominal metastasis exhibited a poor survival time, which may have been due to the lack of effective RT as a local therapy, as there is no effective treatment for abdominal metastasis.

With regard to treatment-related factors, compared with the patients in the <2 years group, the patients in the ≥5 years and ≥2–5 years groups experienced a PR or SD to first-line chemotherapy significantly more frequently. The previous studies of Van Damme *et al* (9) and Giroux Leprieur *et al* (8), as well as the present study, indicated that the response to first-line chemotherapy was a significantly important variable for long-term survival of >2 years.

In the present study, it appears that the various regimens used following progression subsequent to first-line chemotherapy contributed to the extended survival rate, leading to a relatively long MST (14 months) and facilitating the survival of 70 patients (26.1%) for ≥2 years and 22 patients (8%) for ≥5 years. As the study by Hotta *et al* (17) indicated that survival post-progression has become more closely associated with the OS rate by intensive treatment following progression, the current study indicated that chemotherapy subsequent to progression following first-line chemotherapy would be extremely important variables for achieving ≥5 years of survival.

Kaira *et al* (11) found that 10 patients (8%) survived for ≥5 years in a study of 124 patients with advanced NSCLC treated with chemotherapy, the same as the current study. Accordingly, Kaira *et al* concluded that a favorable PS, the presence of adenocarcinoma and the use of EGFR-TKI therapy were significant factors in patients surviving >5 years. Only two of the 10 patients described in the aforementioned study underwent EGFR mutation testing. Therefore, there is no information from the study about the correlation between treatment and EGFR mutation in patients who survived for ≥5 years. In the present study, of the 17 patients in the ≥5 years group who underwent EGFR mutation testing, seven tested positive for drug-sensitive EGFR mutations, two for other types of EGFR mutations and eight for wild-type EGFR. All seven patients with drug-sensitive EGFR mutations were placed in groups A or B. No patients in groups C and D had drug-sensitive EGFR mutations. This study showed a clear correlation between treatment and EGFR mutation status in a higher number of patients than the study by Kaira *et al* (11). In the present study, seven patients with EGFR drug-sensitive mutations achieved ≥5 years of survival. Since patients with drug-sensitive EGFR mutations would potentially survive for ≥5 years, it is extremely important to select patients for EGFR-TKI therapy by screening for drug-sensitive EGFR mutations. Notably, in the present study certain patients without EGFR drug-sensitive mutations would have achieved ≥5 years of survival by continuing long-term effective cytotoxic agent treatments.

The limitations of the study were that it was retrospective, conducted in a single center and normally, patients with improved prognoses would have received more aggressive treatments. Furthermore, patients with a longer survival time would have received multiple regimens and long-term treatments. The study consisted of consecutive and unselected patients at the Osaka Prefectural Medical Center for Respiratory and Allergic Diseases, and reflected the daily practical treatments of NSCLC patients with distant metastasis who received first-line chemotherapy between 2004 and 2007.

In conclusion, achieving ≥5 years of survival in NSCLC patients with distant metastasis was associated with the never-smoked status, response to first-line chemotherapy, EGFR-TKI therapy according to genetic testing, continuing effective cytotoxic regimens and RT as local therapy.

## Figures and Tables

**Table I:** Univariate and multivariate analysis of prognostic factors (n=268).

		Univariate		Multivariate	
			
Variables	n (%)	P-value	HR (95% CI)	P-value	HR (95% CI)
Gender		0.0003	1.72 (1.278–2.314)	0.577	1.106 (0.776–1.577)
Male	186 (69)				
Female	82 (31)				
Age, years		0.51	0.92 (0.703–1.192)		
>66	137 (51)				
<66	131 (49)				
Smoking status		1.2×10^−7^	2.23 (1.659–3.409)	0.0009	1.86 (1.290–2.675)
Never	87 (32)				
Ever	181 (68)				
PS		4.1×10^−5^	2.02 (1.442–2.822)	5.6×10^−6^	2.21 (1.564–3.106)
0–1	213 (79)				
2–3	55 (21)				
Histology		0.0091	1.59 (1.123–2.261)		
Ad	220 (82)				
Non-Ad	48 (18)				
Relapse following surgery		0.0017	1.77 (1.238–2.519)	1.48×10^−5^	2.28 (1.571–3.317)
Yes	49 (18)				
No	219 (82)				
Number of metastatic organs		0.0460	1.32 (1.005–1.732)		
1	161 (60)				
≥2	107 (40)				
Metastatic sites					
Intra-thoracic organs		0.0007	0.607 (0.456–0.810)	0.0009	0.593 (0.435–0.808)
Yes	185 (69)				
No	83 (31)				
Abdominal organs		2.6×10^−5^	2.07 (1.474–3.649)	0.0006	1.88 (1.311–2.689)
Yes	55 (21)				
No	213 (79)				
Brain		0.48	1.13 (0.807–1.584)		
Yes	55 (21)				
No	213 (79)				
Bone		0.0592	1.38 (0.997–1.899)		
Yes	58 (22)				
No	210 (78)				
Lymph nodes (distant)		0.6	1.12 (0.741–1.684)		
Yes	29 (11)				
No	239 (89)				

Ad, adenocarcinoma; HR, hazard ratio; CI, confidence interval; PS, performance status.

**Table II:** Patient characteristics according to survival duration.

Variables	≥5 years, n	≥2–5 years, n	<2 years, n
Gender			
Male	9	32	145
Female	13^a^	16	53
Smoking status			
Never	17^a^,^b^	18	53
Ever	5	30	145
PS			
0 or 1	22^a^	43^c^	148
2 or 3	0	5	50
Histology			
Ad	22	41	157
Non-ad	0	7	41
Relapse following thoracic surgery			
Yes	7^a^	16^c^	26
No	15	32	172
Metastatic site			
Intra-thoracic organs			
Yes	20^a^	38^c^	127
No	2	10	71
Abdominal organs			
Yes	1	3^c^	51
No	21	45	147
EGFR mutation			
Wild-type	8	9	13
Exon19 deletion	5	10	3
Exon21L858R	2	1	0
Other	2	0	2
Unknown	5	28	180

≥5 years, n=22; ≥2–5 years, n=48; <2 years, n=198.

a ≥5 years vs. <2 years, P<0.05;

b ≥5 years vs. ≥2–5 years, P<0.05;

c ≥2–5 years vs. <2 years, P<0.05.

Ad, adenocarcinoma; PS, performance status; EGFR, epidermal growth factor receptor; ≥5 years, patient group who survived ≥5 years; ≥2–5 years, patient group who survived ≥2 years, but <5 years; <2 years, patient group who survived <2 years.

**Table III:** Treatment efficacy, duration of EGFR-TKI therapy and number of cycles of cytotoxic agents according to survival duration.

Variables	≥5 years	≥2–5 years	<2 years
Response to first-line chemotherapy, n			
PR or SD	20^a^	39^b^	103
PD or NE	2	9	95
Number of regimens			
Median	3.5^a^	4^b^	2
Range	1–13	1–9	1–7
EGFR-TKI therapy			
Number of patients, n (%)	14 (63.6)^a^	27 (56.3)^b^	59 (29.8)
Total treatment months			
Median	40.1^a^,^c^	11.0^b^	1.6
Range	0.4–93.6	0.2–60.2	0.1–11.6
Cytotoxic agent therapy			
Number of patients, n (%)	19 (86.4)	47 (97.9)	187 (94.4)
Total treatment cycles, n			
Median	36^a^,^c^	16^b^	4
Range	4–92	1–52	1–18
Palliative RT			
Number of patients, n (%)^d^	13 (59.1)	30 (62.5)	99 (50.0)
Whole brain RT, n	3	10	25
SRS to brain metastasis, n	5	7	25
RT to bone metastasis, n	5	20	56
RT to other metastasis, n	4	0	19

≥5 years, n=22; ≥2–5 years, n=48; <2 years, n=198.

a ≥5 years vs. <2 years, P<0.05;

b ≥2–5 years vs. <2 years, P<0.001;

c ≥5 years vs. ≥2 and <5 years, P<0.05.

d Total number of patients who received at least one form of palliative RT (whole brain RT, SRS to brain metastasis, RT to bone metastasis, or RT to other sites of metastasis).

EGFR-TKI, epidermal growth factor receptor-tyrosine kinase inhibitor; RT, radiotherapy; SRS, stereotactic radiosurgery; PR, partial response; SD, stable disease; PD, progressive disease; NE, not evaluable; ≥5 years, patient group who survived ≥5 years; ≥2–5 years, patient group who survived ≥2 years, but <5 years; <2 years, patient group who survived <2 years.

**Table IV:** Driver mutations in and treatment of patients who survived ≥5 years according to response to EGFR-TKI and cytotoxic agent.

A, Responders to mainly EGFR-TKI therapy			

Case	Driver mutation	Cytotoxic agents, cycles	EGFR-TKIs, months
1	EGFR: L858R	0	93.6
2	-	0	67.4
3	EGFR: del19	5	51.1
4	-	9	61.4
5	EGFR: del19	0	67.1

B, Responders to both EGFR-TKI and cytotoxic agent therapy			

Case	Driver mutation	Cytotoxic agents, cycles	EGFR-TKIs, months

6	EGFR: del19	54	38.0
7	-	29	42.3
8	EGFR: L858R	43	22.0
9	EGFR: del19	28	25.1
10	EGFR: del19	16	49.5

C, Responders to mainly cytotoxic agent therapy			

Case	Driver mutation	Cytotoxic agents, cycles	EGFR-TKIs, months

11	Triple negative	87	9.2
12	ALK translocation	92	5.5
13	-	64	3.2
14	Triple negative	52	0.0
15	Triple negative	58	0.0
16	-	36	0.0
17	EGFR: del19/T790M	43	0.4
18	Triple negative	68	0.0

D, Patients with indolent tumors			

Case	Driver mutation	Cytotoxic agents, cycles	EGFR-TKIs, months

19	Triple negative	10	0.0
20	Triple negative	10	0.0
21	EGFR: L861Q	4	0.0
22	Triple negative	10	0.0

EGFR-TKI, epidermal growth factor receptor-tyrosine kinase inhibitor; ALK, anaplastic lymphoma kinase; triple negative, negative for EGFR mutation, ALK translocation and K-ras mutation.
